# The Role of Interventional Irisin on Heart Molecular Physiology

**DOI:** 10.3390/ph15070863

**Published:** 2022-07-14

**Authors:** Foad Alzoughool, Mohammad Borhan Al-Zghoul, Bayan Y. Ghanim, Michael Gollob, Nasir Idkaidek, Nidal A. Qinna

**Affiliations:** 1 Department of Medical Laboratory Sciences, Faculty of Applied Medical Sciences, The Hashemite University, Zarqa 13133, Jordan; foad@hu.edu.jo; 2 Faculty of Health Sciences, Higher Colleges of Technology, Fujairah Women’s College, Fujairah P.O. Box. 25026, United Arab Emirates; 3 Basic Veterinary Sciences, School of Veterinary Medicine, Jordan University of Science and Technology, Irbid 22110, Jordan; alzghoul@just.edu.jo; 4 University of Petra Pharmaceutical Center (UPPC), University of Petra, Amman 11196, Jordan; bayan.ghanim@uop.edu.jo; 5 Peter Munk Cardiac Centre, Toronto General Hospital, University of Toronto, Toronto, ON M5G 2N2, Canada; michael.gollob@uhn.ca; 6 Department of Pharmaceutics and Pharmaceutical Technology, Faculty of Pharmacy and Medical Sciences, University of Petra, Amman 11196, Jordan; nidkaidek@uop.edu.jo; 7 Department of Pharmacology and Biomedical Sciences, Faculty of Pharmacy and Medical Sciences, University of Petra, Amman 11196, Jordan

**Keywords:** irisin, myocytes, gene expression, heart physiology

## Abstract

Irisin, encoded by the FNDC5 (fibronectin type III domain containing 5) gene, is a novel myokine that has been implicated as an essential mediator of exercise benefits. Effects of irisin on heart physiology is still ambiguous. This study aimed to evaluate the impact of exogenous administration of irisin on heart physiology and the pharmacokinetic profile of pump-administered irisin. To do so, Sprague Dawley rats were implanted with an irisin-loaded osmotic pump (5 μg/kg/day) for 42 days, and other animals were administered with single bolus subcutaneous injections of irisin (5 µg/kg). Body weights and blood samples were collected weekly for 42 days for serum irisin quantification and histopathology. Clinical biochemistry analyses were performed. Heart mRNA expression was assessed in 26 selected genes. Chronic interventional exogenous irisin significantly reduced body weight without affecting the heart myocyte size and significantly reduced creatine kinase enzyme level. Blood CBC, serum biochemistry, and heart morphology were normal. Gene expression of FNCD5, Raf1, CPT1, IGF-1, and CALCIN, encoding for heart physiology, increased while PGC1, Nox4, and Mfn1 significantly decreased. Nevertheless, irisin increased the expression of cardioprotective genes and inhibited some genes that harm heart physiology. Administration of irisin promotes myocardial functions and could be translated into clinical settings after preclinical profiling.

## 1. Introduction

Physical exercise is correlated with hemodynamic changes and alters the loading conditions of the heart. The major components of cardiac output—stroke volume and heart rate—are the main hemodynamic features that change an athlete’s heart [1]. These adaptive changes reflect positive changes that allow the heart to increase its capacity to supply blood and oxygen to exercising tissues. An athlete’s heart mostly has a benign increase in cardiac mass, with particular circulatory and cardiac morphological alterations that exemplify a physiological adaptation to a comprehensive training program [2,3]. Many genes seem to be engaged in physical performance and cardiovascular responses. MEK1 transgenic mice showed spectacular cardiac function by stimulating a physiological hypertrophy response associated with the boosted cardiac function [4]. Insulin-like growth factor 1 (IGF-1) has been shown to improve the myocardial function of normal adult rats [5]. Expression of peroxisome proliferator-activated receptor γ coactivator 1α (PGC1α) reduces pathological remodeling of older people’s hearts and could thereby contribute to the beneficial effects of exercise on cardiac function in ageing [6].

Irisin is a novel hormone secreted by myocytes and has been suggested to mediate some of the beneficial effects of exercise [7]. It is a muscle-derived myokine released to the circulation from the fibronectin type III domain-containing protein 5 (FNDC5) after the cleavage of its extracellular portion. FNDC5 has been proposed to induce browning of subcutaneous adipocytes and thermogenesis by increasing uncoupling protein 1 (UCP1) levels both in culture and in mouse models [8]. It is hypothesized that FNDC5 induces the differentiation of a subset of white adipocytes into brown fat and mediates the beneficial effects of exercise on metabolic homeostasis and energy expenditure [9]. It has been demonstrated in the literature that, upon exercise stimulation and through PGC1α, the expression of FNDC5 in muscle is enhanced and subsequent irisin is secreted, inducing the stimulation of thermogenesis genes in certain adipocytes [10]. Recently, it has been reported that circulating irisin is increased transiently by an acute attempt of exercise, indicating that its concentration correlates with exercise intensity. Moreover, supplementing cultured human cells with exogenous irisin results in the regulation of adipocyte browning, muscle growth, and metabolism [8,11,12]. As exercise is an excellent therapeutic intervention for pathologies such as obesity, type 2 diabetes, cardiovascular disease, and neurodegeneration, irisin has been considered a potential therapeutic candidate to mimic the physiological effects of exercise and treat many diseases [13,14].

In the present study, we examined the potential effects of interventional exogenous irisin administration (chronic model) on gene expression of proteins associated with adaptive physiological cardiac changes and myocardial health. In addition, a superimposition simulation model of repetitive subcutaneous administrations of irisin was generated and compared to a pump-released experimental model. The potential cardiometabolic impact of chronic irisin administration is also discussed. 

## 2. Results

### 2.1. General Clinical Observations

#### Body Weights

**Figure 1:**
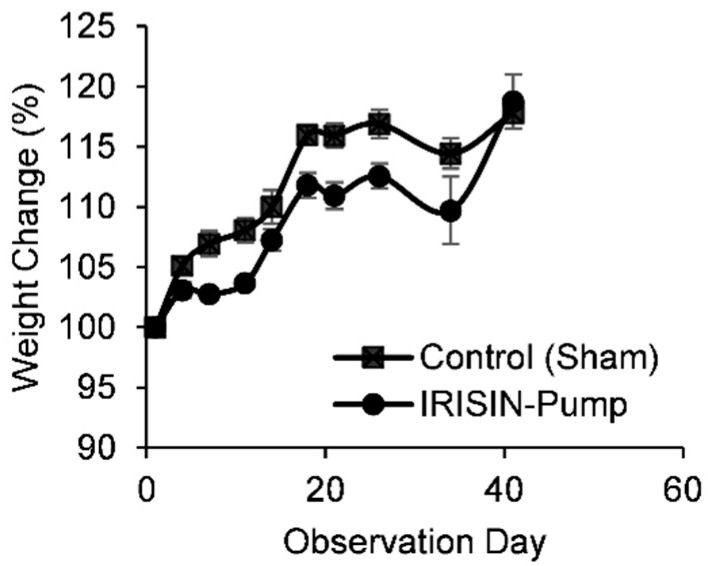
Percent weight change of rats administered with irisin via the osmotic pump (*n* = 10). Irisin reduced body weight up to 5% from day 7 and up to day 34 of administration. For example, average body weight dropped from around 209 g to 196 g at day 21 of administration.

Changes in percent bodyweight of study animals are shown in Figure 1. A decrease in percent change of body weight in animals treated with irisin was considered clinically significant compared to animals of the non-treated group. Irisin reduced body weight up to 5% from day 7 and up to day 34 of administration. For example, the average body weight dropped from around 209 g to 196 g at day 21 of administration.

### 2.2. CBC and Clinical Biochemistry

**Figure 2:**
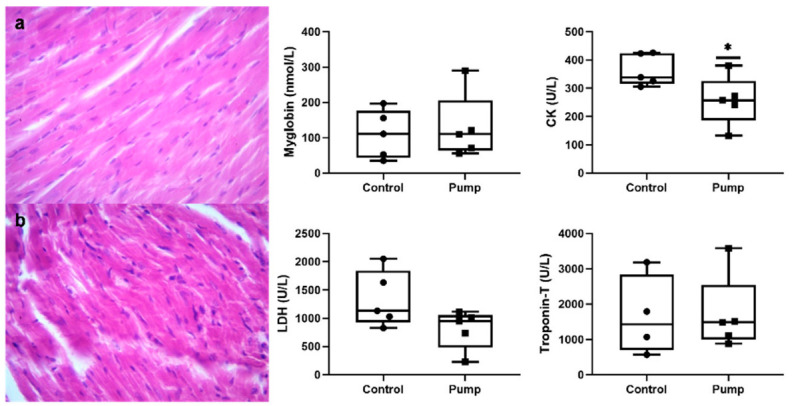
Morphological and functional evaluation of cardiac and hind muscle of irisin-treated rats. Cardiac tissue sections of (**a**) control and (**b**) irisin (pump)-treated animals (×40). Levels of muscle activity parameters including myoglobin; CK: creatine kinase; LDH: lactate dehydrogenase, and troponin-T were analyzed in the serum of control and irisin-treated animals. * *p*-value < 0.05.

Blood analysis for CBC is shown in Table 1. Changes in CBC parameters were considered clinically insignificant and indicated no alteration due to irisin concentrations. Levels of blood monocyte count and platelet distribution width were affected by irisin administration. However, the correlation between the parameters and irisin accumulation has not been reported earlier in the literature. Most of the serum biomarkers were clinically normal and not triggered by irisin administration. However, levels of creatine kinase were significantly decreased in rats administered with irisin as shown in Table 1 and Figure 2. Nevertheless, the drop in LDH levels was considered statistically insignificant.

### 2.3. Histopathology

As shown in Figure 2, sections of cardiac muscle tissue were evaluated. No signs of morphological changes were observed in irisin-treated animals in heart tissue sections compared to control.

### 2.4. Quantification of Serum Irisin

**Figure 3:**
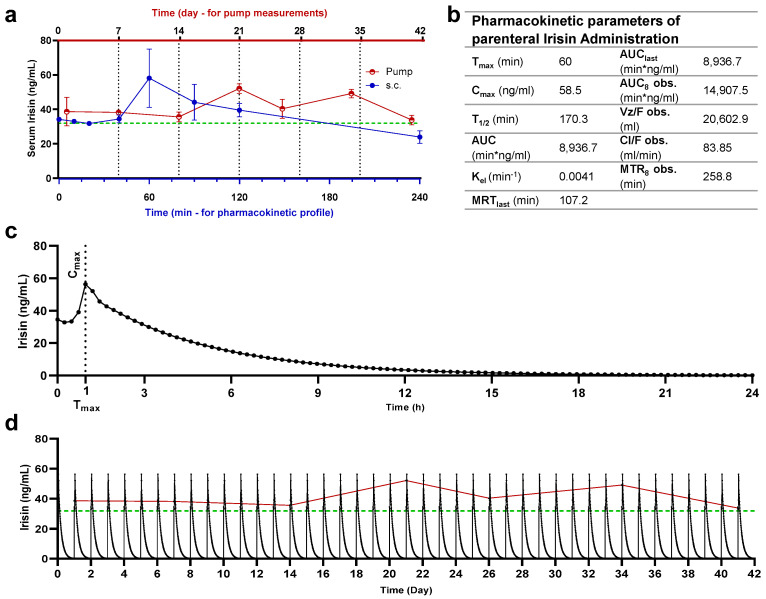
Relative and simulation-generated pharmacokinetic profiles of irisin. (**a**) Serum sampling and irisin quantification of irisin administered via the osmotic pump (upper *x*-axis—red) and subcutaneous route (bottom *x*-axis—blue) in Sprague Dawley rats, as well as baseline levels measured in control animals (dashed line—green). Irisin quantification was made at specific time intervals after administration: weekly during 42 days of osmotic pump release for rats receiving irisin via a pump and 4 h from the subcutaneous injection (min); (**b**) pharmacokinetic parameters of irisin in Sprague Dawley rats receiving irisin via the subcutaneous route; (**c**) simulated kinetic profile of single-dose subcutaneous administration; (**d**) repeated for 42 days with pump readings plotted with baseline levels of control animals (dashed line—green) and pump-released irisin levels (continuous line—red).

Serum irisin levels of study animals are shown in Figure 3. A pharmacokinetic profile was developed by analyzing serum irisin levels in animals after subcutaneous administration of irisin. Peak serum irisin level was detected after 1 h from dosing at 58.5 ng/mL.

Levels of serum irisin were determined for intervals of the dosing periods. For animals receiving irisin via the osmotic pump, sampling was conducted at intervals from 7 days to 42 days. As for animals receiving irisin as subcutaneous bolus injections, sampling was conducted at intervals of up to 4 h. In comparison to levels of irisin in non-treated rats, alterations of increase in irisin were noted on multiple days throughout the dosing period, thus confirming the consistent release of irisin from the osmotic pump.

The administration of irisin via an osmotic pump consistently released irisin throughout the 42 days at levels higher than the baseline. Baseline levels were estimated as 24 ng/mL in control groups and after 4 h from subcutaneous administration. Simulation data were built using a subcutaneous pharmacokinetics profile and compared with the pump-released irisin profile. Daily administration of irisin is needed to establish a profile similar to the pump.

### 2.5. Gene Expression Levels

**Figure 4:**
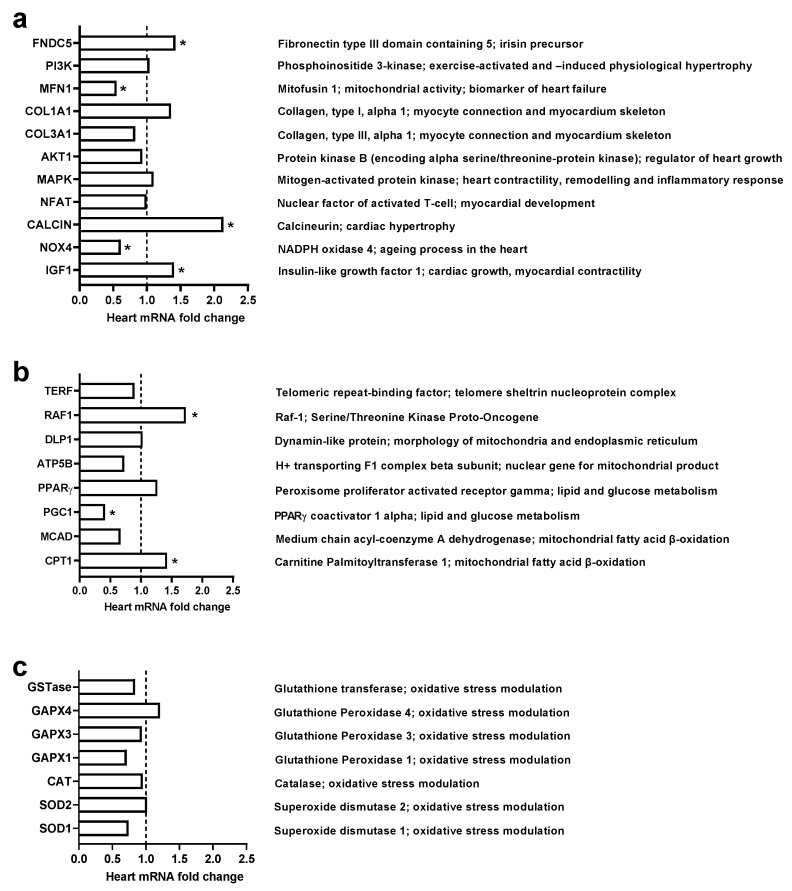
Effect of chronic administration of exogenous irisin (5 μg /kg/day) for 42 days on heart mRNA levels (*n* = 5). (**a**): Genes encoding for myocyte homeostasis and activity; (**b**): genes encoding for metabolism and mitochondrial activity; (**c**): genes encoding against oxidative stress. * *p* < 0.05 in comparison to the control group.

Our study investigated the potential change in gene expression of 28 selected heart genes (shown in Figure 4). Chronic administration of exogenous irisin (5 μg/kg/day) for 42 days significantly changed the gene expression level of 9 out of the 28 studied genes. Amongst genes responsible for cardiac muscle integrity and physiology (Figure 4a), irisin significantly enhanced the expression of FNCD5, a precursor of irisin. Furthermore, it inhibited the expression of NOX4 and Mfn1. Irisin significantly upregulated the expression of CALCIN and IGF1 (Figure 4a). Regarding genes modulating cellular functions and survival, irisin significantly increased the expression of oncogene RAF-1 and triggered b-oxidation genes, namely CPT1 and CPG1 (Figure 4b). Moreover, genes expressed against oxidative stress were found unchanged (Figure 4c).

## 3. Discussion

Since 2012, the discovery of irisin has raised questions about its role in longevity and maintaining a healthy life through its capacity of mimicking exercise action and offering a novel therapeutic intervention for many ailments such as obesity, type 2 diabetes, cardiovascular disease, and neurodegeneration. Several previous animal studies reported the valuable role of irisin in metabolic homeostasis and maintaining body mass index (BMI) [15], and against brain ischemia [16], ischemic reperfusion injury [17], and diabetes [18]. Although skeletal muscle is an abundant resource of irisin, the heart is also reported to secrete the myokine [19], suggesting the vital role of irisin in myocardial physiology. In addition, a study reported that irisin regulates cardiac activity in zebrafish, where exogenous irisin treatment increased diastolic volume, heart rate, and cardiac output, whereas irisin knockdown yielded opposing effects on cardiovascular functions [20].

In this study, chronic administration of interventional exogenous irisin for 42 days showed a valuable role in reducing body weight without affecting the heart myocyte size. At the day of termination, change in body weight was no longer observed, and that could be linked to the uptake of irisin in the pump, demonstrating a direct effect of irisin on reducing body weight. Nevertheless, such observation warrants further investigation in the impact of irisin on adipocyte and skeletal muscle mass. In the current study, irisin significantly reduced creatine kinase and thus reducing the chance of heart muscle damage. In addition, irisin increased the expression of some genes protecting cardiac myocytes and cardiac function such as FNDC5, CPT1, IGF-1, and Calcin while inhibiting the expression of some genes that harm heart physiology, such as PGC1, NOX4, and Mfn1.

The presented pharmacokinetic and protein experiments confirmed a consistent release of irisin from the osmotic pump. It seems that the exogenous interventional irisin has no toxic effect in the animal model study, i.e., no clinical significance was detected on CBC parameters after irisin administration. The exception is platelet distribution width (PDW), where the change was considered statistically significant but of negligible clinical impact since the platelet count was not affected. Moreover, most serum biomarkers were considered clinically normal and untriggered upon administration of irisin, especially ALT, AST, and ALP. However, creatine kinases and LDH were decreased in rats administered with irisin, thus supporting the beneficial effects of irisin on health. Previous research did not report any toxic effects or inverse impact on animal health [21]. The negative correlation between serum irisin concentrations and creatine phosphokinases is documented in the literature and proposed as an accurate biomarker along with irisin in cases of myocardial pathologies [22,23]. Despite the decrease in LDH levels upon irisin administration, statistically and clinically, it is considered insignificant. Thus, the correlation between LDH and irisin cannot be confirmed yet. Serum levels of troponin were unaffected by the increase of serum irisin and were found to be comparable to control levels. According to the recent literature, the correlation between irisin levels and troponin is negative, as several studies confirm its decrease upon elevation in serum irisin levels [24,25]. The presented results question the reliability of troponin as a biomarker of cardiac health [26] and raise attention to a circumstantial correlation to the concentrations of irisin in subjects.

The present study reported a considerably clinically significant decrease in percent change of body weight in animals treated with irisin compared to animals of the non-treated group. A previous study in obese mice showed a significant decrease in BMI [15], while another suggests that irisin can potentially prevent obesity by stimulating the expression of genes specific to white adipocyte tissue-browning [12]. These studies support our finding of reduced body weight in rats treated with irisin. Our result might also be novel, as irisin reduces body weight without exposing the rat to any exercise. This supports the hypothesis that irisin might be a therapeutic alternative to exercise, especially for those who cannot engage in any physical activity.

Our present study investigated the level of heart gene expression of rats exposed to continuous exogenous irisin for 42 days (0.15 µL/h = 5 µg/day). The results showed a significant increase in essential genes that have a substantial role in cardiac energy and cardiac myocyte contractility and hypertrophy, such as IGF-1, Raf1, CPT1, and CALCIN. On the other hand, irisin inhibited the expression level of many genes that pose potential harm to heart physiology, such as NOX4 and Mfn1. Together, these results suggest an opportunity to develop irisin as a novel pharmaceutical compound for a healthy heart.

Our result showed that chronic intervention of exogenous irisin significantly increased the expression level of the IGF-1 gene. According to a previous experimental study, IGF-1 inhibits the progression of cardiomyopathic disease in a transgenic mouse model of heart failure, suggesting that heart failure may benefit from early treatment with interventional IGF-1 [27]. This adds extra value to our study, as interventional irisin increases the expression of the IGF-1 gene, which might protect against heart failure. Another descriptive study investigated the partial deficiency of IGF-1 and its influence on the heart and coronary circulation before and after ischemia–reperfusion (I/R). The authors showed that IGF-1 partial deficiency is correlated with decreased contractility, angiotensin II sensitivity, and interstitial fibrosis, as well as changes to gene expression involving calcium dynamics and cardiac physiology [28]. This also supports our suggestion that irisin might have a myocardial-protective role via increased gene expression of IGF-1.

Calcineurin (CN) is a calcium- and calmodulin-dependent protein phosphatase that provides an essential contractual point for coordination between two fundamental modes of intracellular communication: protein phosphorylation and calcium signaling cascade [29]. It is well-known that calcineurin primarily acts through the nuclear factor of activated T-cell (NFAT) family of transcription factors for pathological cardiac remodeling; however, calcineurin also has an essential role in the development and homeostasis in the adult heart [30]. The calcineurin/NFAT transcriptional pathway is not the only signal initiated by calcineurin in the setting of pathological remodeling. Our results showed a significant increase in calcineurin gene expression despite the absence of change in gene expression of NFAT transcriptional factor gene level, suggesting that irisin might be essential for non-pathological heart development. More studies are needed to identify the exact role of irisin in calcineurin pathways.

Fatty acids and acyl-CoAs cannot enter the mitochondrial membrane unless they conjugate with creatinine. In the heart, long-chain fatty acids form a high-energy ester bond with carnitine facilitated by carnitine palmitoyltransferase 1 (CPT-1), located in the inner aspect of the outer mitochondrial membrane [31]. Activation of CPT1 leads to an increase in the mitochondrial import and oxidation of long-chain acyl-CoA fatty acids in muscle [32]. Increasing fatty acid beta-oxidation in the heart and skeletal muscle may reduce and prevent cytoplasmic lipid accumulation and decrease insulin resistance [33]. As our results showed, a significant increase in CPT1 gene expression in response to chronic interventional exogenous irisin might lead us to conclude that irisin may help prevent cytoplasmic lipid accumulation in cardiac myocytes for better cardiac function.

Several previous studies on Raf-1 knockout mice have suggested that Raf-1 plays an essential role in preventing apoptosis [34,35]; however, induction of apoptosis leads to the development and progression of cardiac dysfunction [36]. Nevertheless, previous studies also reported that Raf-1 promotes cardiomyocyte survival [36]. Our investigation of heart Raf-1 gene expression in response to irisin reported that irisin significantly increased the gene expression of Raf-1, which is in line with previous studies and leads us to suppose that irisin might have a very beneficial effect by preventing apoptosis that might harm cardiac function.

Results showed not only increased expression of the genes that positively impact heart function and development, but also inhibition of the expression of genes that might be harmful to cardiac physiology, such as Nox4 and Mfn1. This suggests that irisin might have a role in protecting the heart against I/R via inhibition of the gene expression of Nox4. We recommend further investigation on the interventional exogenous irisin for the treatment of I/R. Previously, it was suggested that Nox4 plays a very important role in mediating oxidative stress and myocardial injury after I/R [37]. Interestingly, it was reported that cardiac and systematic knockdown of Nox4 significantly reduced infarct size and area at risk after the ischemia–reperfusion challenge [37]. Inhibition of Nox4, which is expressed in cardiomyocytes by a selective inhibitor (GLX481304), reduced the generation of free reactive oxygen species in mouse cardiac muscle and enhanced the cell contractile function, improving the whole heart after a hypoxic/ischemic–reperfusion challenge [38].

Irisin inhibits Mfn1 gene expression in heart tissue, and according to previous studies, deficient Mfn1 hearts are protected against acute myocardial infarction due to impaired mitochondria/SR tethering [39]. It was shown that acute ablation of both cardiac Mfn1 and Mfn2 makes the heart resistant to acute myocardial infarction [39]. This adds value to our result, as irisin might also protect the heart against myocardial infarction by inhibition of Mfn1 gene expression.

As concluded in a systematic review, two out of eleven studies report that chronic exercise induces the expression of both genes. However, nine studies reported no change in gene expression after chronic exercise [40]. On the contrary, some research revealed that an overexpression in heart PGC-1α composes acute survival and fails to improve cardiac function during chronic pressure overload in mice [41]. In addition, chronic exercise does not induce or change the expression of either PGC1 or FNDC5. This is consistent with the presented findings, as the chronic intervention of exogenous irisin significantly inhibits the expression of PGC-1. However, it increased the expression of FNDC5, which may raise a new question of a possible positive feedback mechanism where irisin may induce the expression of FNSC5, the precursor of irisin protein.

Despite the documented benefits of irisin on cardiac health and clinical management, a controversy around its impact on a coronary heart disease patient is still unsolved. Moreover, during heart failure, irisin was reported to influence cellular metabolism, mitochondrial energetics, and heart failure prognosis. The benefits of irisin on muscle and cardiac health are multiple, yet some controversies are still raised considering its safety in clinical settings. By contrast, high levels of irisin in myocardial infarction might be associated with more cardiovascular risks. Findings reported by Hsieh et al. showed that serum concentrations of irisin were increased in patients post-ST-elevation myocardial infarction [42]. However, the link between the presence of high levels of irisin during or after cardiovascular pathologies is still ambiguous and the controversial role of irisin in the clinical management of heart diseases still needs depth identification of its molecular mechanisms. Nevertheless, due to the dual effects of irisin on cardiovascular physiology, irisin could be categorized as a critical therapeutic target in cardiovascular diseases.

The impact of irisin on cardiovascular disease has also been reported as effective for diabetic subjects, showing a reduction in the complications that may arise in such sensitive patients. Multiple studies on diabetic patients concluded that serum irisin levels are associated with enhanced prognosis in patients with cardiovascular comorbidities. A study by Khorasani et al. showed that irisin levels were relatively lower in diabetic patients with cardiovascular complications in comparison to uncomplicated diabetic patients [43]. On the contrary, other studies concluded that increased irisin levels mediate cardioprotection in diabetic subjects and inhibit myocardial apoptosis, implicating irisin as a potential therapeutic intervention during diabetic cardiomyopathy [44,45,46].

## 4. Materials and Methods

### 4.1. Experimental Design

Twenty adult female Sprague Dawley rats with an average weight of 250 ± 40 g were supplied by the Animal Research Unit of the University of Petra Pharmaceutical Center (UPPC), University of Petra (Amman, Jordan). Female animals were selected due to the complexity of the female endocrine system and for being preferable in toxicity assessment if any. Rats were maintained under climate-controlled temperatures (22–24 °C), humidity (55–65%), and a 12-h light/dark cycle.

### 4.2. Pump Implantation and Irisin Administration

After acclimatization, rats were randomized into two groups (*n* = 10): a sham group that received a plain surgery and an irisin-treated group that received an irisin-loaded osmotic pump. The dorsal area of the rats was shaved using an electronic clipper and prepared for surgery. Animals were placed over a surgical platform (Kent Scientific, CT, Torrington, USA) and anesthetized by isoflurane (Hikma Pharmaceuticals, Amman, Jordan) (5% induction and 2.5% maintenance) carried by oxygen (Dual flow oxygen concentrator, Hebei, China) using a low-flow anesthesia system (SomnoSuite, Kent Scientific, CT, Torrington, USA). After reaching the surgical depth of anesthesia, povidone–iodine was applied for preoperative skin preparation, and an incision was created for the implantation of the pump (ALZET Osmotic Pump 2006, California, USA). The pump was filled with 150 μL of 0.25 μg/μL recombinant human fibronectin type III domain-containing protein 5 (FNDC5) (CusaBio, Houston, TX, USA) reconstituted with PBS (Hyclone, Logan, UT, USA). The pump, as claimed by the manufacturer, releases 0.15 μL/h, thus delivering a dose of 5 μg/kg/day. Incisions were closed with surgical staple sutures and observed closely during the first day and daily throughout the study for mortality, general assessment, and any abnormal clinical signs.

### 4.3. Parenteral Irisin Administration and Quantification

Three female Sprague Dawley rats were acclimatized and prepared for validating irisin delivery and quantification in the serum. Animals were restrained and administered bolus subcutaneous injections of irisin (5 µg/kg). Afterwards, animals were subjected to blood sampling from the tail tip at specific time intervals (0, 10, 20, 40, 60, 90, 120, 240 min) and serum was collected for quantification using ELISA analysis.

### 4.4. Sampling

Body weights and blood samples were collected weekly for 42 days. Less than 100 µL blood was withdrawn from the ocular sinus using a heparinized capillary tube into plain tubes. Then, serum was collected for hormone quantification and biochemical analysis through centrifuge at 200× *g* for 10 min. At the day of study termination, specifically day 42, animals were subjected to euthanasia by 5% isoflurane, and blood from the ocular sinus was collected into EDTA tubes and plain tubes. Whole blood in EDTA tubes was tested for CBC within 24 h and serum separated following centrifugation was stored at −20 °C for further biochemical analysis at MedCare Labs (Madaba, Jordan). The heart was collected for histopathology and RNA extraction.

### 4.5. Irisin Quantification

According to the manufacturer’s instructions, serum irisin levels were quantified using the irisin ELISA kit (CusaBio, Houston, TX, USA).

### 4.6. Superposition Principle Simulation Modelling

Simulation of daily administration of exogenous irisin was based on data acquired through parenteral administration. Data were processed considering a dose of 1.25 µg to 250 g rat mass every 1440 min (24 h). Analysis was run using WinNonlin Noncompartmental Analysis Program (Version 5.2). Non-parametric superposition methodology was employed to predict the drug concentration in blood or plasma after multiple doses based on concentration data from a single dose. This was achieved by fitting the data based on the principle of superposition, which does not assume any pharmacokinetic (PK) model. To predict the drug concentration resulting from multiple doses, the concentration–time profile of a single dose was processed. Two assumptions about the data are required: independence of each dose–effect and linearity of the underlying pharmacokinetics. The former assumes that the effect of each dose can be separated from the effects of other doses. The latter, linear pharmacokinetics, assumes that changes in drug concentration will vary linearly with dose amount. The required input data are time, dosing regimen, sort variables, and drug concentration after a single dose. The method of computation was set to log without defining the terminal phase.

### 4.7. RNA Isolation and cDNA Synthesis

On day 42 following the administration of irisin, cardiac samples were taken from all animals and snap-frozen on-site with liquid nitrogen to avoid RNA degradation. These samples were then kept at 20 °C in TRI Reagent^®^ solution tubes (Zymo Research Co., California, Irvine, CA, USA). A Bead Ruptor Elite-Bead Mill Homogenizer was used to homogenize tissue samples (OMNI International, Kennesaw, GA, USA). Homogenized samples were then processed with Direct-ZolTM RNA MiniPrep (Zymo Research Co.) and TRI Reagent^®^ to isolate total RNA (Zymo Research Co.). Qubit 4 Fluorometer (Thermo Fisher Scientific, Waltham, MA, USA), BioTek PowerWave XS2 Spectrophotometer (BioTek Instruments, Inc., Winooski, VT, USA), and 1% agarose gel was utilized to quantify and qualify the RNA. Then, cDNA was synthesized for each sample using a high-capacity cDNA reverse transcription kit (Applied Biosystems, Foster City, CA, USA).

### 4.8. Relative Quantitative Real-Time PCR (RT-qPCR)

In a Rotor-Gene Q MDx 5 plex instrument (Qiagen, Hilden, Germany), TB Green™ Premix Ex Taq™ II kit (Takara Bio Inc., Kusatsu, Shiga, Japan) was used. Briefly, the 20 µL reaction mix was prepared from 10 µL of the master mix, 1.2 µL forward primer (4 pmol), 1.2 µL reverse primer (4 pmol), 2 µL cDNA of the sample, and 5.6 µL of nuclease-free water. Cycling parameters were 50 °C for 2 min, 95 °C for 15 min, 40 cycles of 95 °C for 15 s followed by 40 s at 57 °C, and 72 °C for 20 s, with final melting at 95 °C for 20 s. Duplicates from each cDNA were analyzed, fluorescence emission was detected, and relative quantification was computed automatically. The fold changes in gene expression were normalized using GAPDH and β-actin as internal controls, with which the melting curve confirmed the single-target amplification specificity.

The cDNA sequence for each gene utilized in the primer design was obtained from the NCBI’s Nucleotide Database (https://www.ncbi.nlm.nih.gov/nucleotide/; accessed on: 1 October 2021). All primers were designed using IDT PrimeQuest™ Tool version (2.2.3) (http://eu.idtdna.com/PrimerQuest; accessed on: 1 October 2021), Integrated DNA Technologies Inc. (Coralville, IA, USA). The primer sequences are presented in Table S1.

### 4.9. Statistical Analysis

Values with *p*-value less than 0.05 were considered significant. Values are presented as mean ± standard error of mean (SEM). One-way ANOVA followed by post-hoc Tukey’s HSD test was performed to calculate the statistical significance of gene expression, whereas Mann–Whitney non-parametric analysis was used for CBC and Clinical Biochemistry analysis using IBM SPSS Statistics 25, IBM Corporation (New York, NY, USA)

## 5. Conclusions

A chronic intervention model of exogenous irisin in rats showed a valuable role for irisin in reducing body weight without affecting myocyte size and significantly reducing creatine kinase and, thus, heart muscle damage. Irisin also increased the expression of some genes protecting cardiac myocytes and cardiac function and inhibited some genes that harm heart physiology. Our results shed light on the importance of performing further clinical and physiological studies. Improved insight into the role of irisin on heart health and pathophysiology promises to lead to more innovative treatment strategies that will improve care of affected patients.

## Figures and Tables

**Table 1:** Complete blood count (CBC) and serum clinical biochemistry. Data presented as Mean ± SEM; * *p* < 0.05; analyzed using Mann–Whitney non-parametric analysis.

		Control	Irisin-Pump
Complete blood count (CBC)	Leukocytes (WBCs) Count (×10^3^)	2.98 ± 0.35	2.34 ± 0.41
	Red blood cells (RBCs) Count (×10^6^)	5.92 ± 0.95	5.60 ± 0.68
	Hemoglobin (g/dL)	12.84 ± 0.52	13.08 ± 0.41
	Hematocrit %	33.8 ± 5.95	31.36 ± 4.51
	Platelets Count (PCT) (×10^3^)	717 ± 54.58	781.60 ± 208.77
	PCT %	0.53 ± 0.09	0.58 ± 0.08
	Mean cell volume (MCV) (μm^3^)	55.8 ± 1.93	55.20 ± 1.83
	Mean cell hemoglobin (MCH) (pg)	26.22 ± 7.25	24.96 ± 3.42
	Mean cell hemoglobin concentration (MCHC) (g/dL)	48.8 ± 15.83	45.98 ± 7.74
	Red cell distribution width (RDW) %	17.22 ± 2.43	18.22 ± 2.58
	Mean platelet volume (MPV) (μm^3^)	7.44 ± 1.10	8.50 ± 1.49
	Platelet distribution width (PDW) %	13.22 ± 0.33	9.34 ± 1.46 *
	Lymphocytes %	86.42 ± 1.11	82.98 ± 1.05
	Monocytes %	10.32 ± 0.67	11.80 ± 0.93
	Granulocyte %	3.26 ± 0.48	5.22 ± 0.24
	Lymphocytes Count (×10^3^)	2.52 ± 0.31	1.88 ± 0.37
	Monocytes Count (×10^3^)	0.26 ± 0.04	0.24 ± 0.04 *
	Granulocyte Count (×10^3^)	0.20 ± 0.00	0.22 ± 0.02
Clinical Biochemistry			
	Cholesterol (mg/dL)	59.8 ± 5.3	67.8 ± 5.6
	Triglycerides (mg/dL)	53 ± 2.4	51.2 ± 7.3
	High-Density Lipoprotein (HDL) (mmol/L)	16.2 ± 1.7	18.6 ± 1.2
	Low-Density Lipoprotein (LDL) (mmol/L)	33.4 ± 3.9	39 ± 3.1
	Cholesterol/HDL Ratio	3.8 ± 0.4	3.62 ± 0.1
	Very Low-Density Lipoprotein (VLDL) (U/L)	10.2 ± 0.6	9 ± 2.1
	Aspartate-aminotransferase (AST:GOT) (U/L)	105 ± 3.7	96.4 ± 7.4
	Alanine-aminotransferase (ALT:GPT) (U/L)	24.6 ± 1.3	26 ± 2.1
	Alkaline Phosphatase (ALP) (U/L)	26.2 ± 5	30 ± 3.7

## Data Availability

Data is contained within the article.

## Supplementary Materials

The following supporting information can be downloaded at: https://www.mdpi.com/article/10.3390/ph15070863/s1.
