# Supplementary material for: The Role of Interventional Irisin on Heart Molecular Physiology
Source: Pharmaceuticals (Basel). 2022 Jul 14;15(7):863. doi: 10.3390/ph15070863 (PMC9319709; doi:10.3390/ph15070863)
Supplement: Supplementary file 1 [file pharmaceuticals-15-00863-s001.zip › pharmaceuticals-1783056-supplementary.pdf]

Table S1. Primers sequences that are used in the real time qPCR analysis.

The gene	Sequence 5’-3’
PPARG	Forward: CGTGTTCCCGATCACCATATT<br>Reverse: GGTGTCTGTAGTGGCTTGATT
PPARγ	Forward: CTGGCCTCCCTGATGAATAAAG<br>Reverse: AGGCTCCATAAAGTCACCAAAG
SOD-2 2	Forward: TAGAGCCTTTGCCTGTCTTATG<br>Reverse: CAATGTCACTCCTCTCCGAATTA
ATPase	Forward: GGAGATACAGGGCTACAGAAAC<br>Reverse: AGCTCTTGCCACTACTTGTC
MFn1	Forward: GGAGATACAGGGCTACAGAAAC<br>Reverse: AGCTCTTGCCACTACTTGTC
DLP1	Forward: CAACTGGAGAGGAATGCTGAA<br>Reverse: TGTGGACTTGCTGGCATAAT
CPT1b	Forward: TGCACGGCAACTGCTATAA<br>Reverse: ATCTGCCCATGAGTGTTCTG
MCAD	Forward: CCGGGAATACACATCGGAAA<br>Reverse: CCTTAGGCACTCTGACATCTTC
Colla1	Forward: CCAATGGTGCTCCTGGTATT<br>Reverse: GTTCACCACTGTTGCCTTTG
Col3a1	Forward: CAGGCCAATGGCAATGTAAAG<br>Reverse: GCCATCCTCTAGAACTGTGTAAG
TREF1	Forward: CAGAGTTATGTGGAGTGTGTGT<br>Reverse: GTTGGTGGCCTTTGCTTTATC
IGF-1R	Forward: CAATATCACAGACCCGGAAGAG<br>Reverse: CGATACGGTACAGAGTGAAAGG
PI3K	Forward: GTATCAAGTCGTGGTGGGAAA<br>Reverse: GTCTTGCCGTAGAGTCCAAATA
Raf-1	Forward: GTGATGCTGTCTACTCGGATTG<br>Reverse: GAGTTGGGTCAACCACCTTTA
AKT1	Forward: CGCTACTTCCTCCTCAAGAATG<br>Reverse: CTGACATTGTGCCACTGAGA
MAPK	Forward: GGAGGATAGCAACATGGAGAAA<br>Reverse: AGACTCTAACCGGACCACTAA
SERCA	Forward: AAAGTGGAGCGGGCTAATG<br>Reverse: GGGAGCAGTAGACAGACATAGA
NFAT	Forward: GGACAGCCAACCTCAAGTAAA<br>Reverse: CCCTGCTGGAACAAGCTATT
CALCIN	Forward: TCCAGGTGTTGAAGATGATGG<br>Reverse: CCCGTCCTTATCTGCGTTTAT
SOD1	Forward: CTCAGGAGAGCATTCCATCATT

	Reverse: CTTCCAGCATTTCCAGTCTTTG
SOD2	Forward: GTGACCTGTAGGAGTGAGTAGA<br>Reverse: GCTCTCCTGAGGTCTGTTTAAG
atalase	Forward: CTCAGGTGCGGACATTCTATAC<br>Reverse: GACCGCTTTCCTCTGAATGA
GAPX2	Forward: ACCTTCCAGACCATCAACATC<br>Reverse: CTTGGAGAAACAGTCGGAGATT
GAPX1	Forward: CCATGACGGTGTTTCCTCTAA<br>Reverse: GTAAAGGCATCGGGAATGGA
GAPX3	Forward: GAGCGGTACCATCTACGAGTAT<br>Reverse: GCTACGTTGACAAAGAGGATGT
GAPX4	Forward: GGCCTGGAAGTGGAGTTAATAG<br>Reverse: CAGTGGTTAGAGGAACGAAGTG
GSTase	Forward: CCGTCACCCTCTGATTGATTTA<br>Reverse: TCCTGATTTCTCTGCTCCTTTC
NOXA1	Forward: AGATGGCCGTGTTGGTATTT<br>Reverse: ATTGCTAGTGCTGTTCTCCTG
GST	Forward: GCCGGAGAGGTTGGTAAATAG<br>Reverse: TCAGCTCCCTTTCCACTTTG
NOX4	Forward: TTCTGGACCTTTGTGCCTATAC<br>Reverse: CCATGACATCTGAGGGATGATT
GAPDH	Forward: GGAGAAACCTGCCAAGTATGA<br>Reverse: TTGAAGTCACAGGAGACAACC
β-Actin	Forward: ACAGGATGCAGAAGGAGATTAC<br>Reverse: ACAGTGAGGCCAGGATAGA
